# The *DWARF27* Gene from Wintersweet (*Chimonanthus praecox*) Encodes an All-Trans/9-cis-β-Carotene Isomerase, Which Regulates Shoot Branching in *Arabidopsis*

**DOI:** 10.3390/plants15121926

**Published:** 2026-06-22

**Authors:** Xia Wang, Yan Zheng, Rong Han, Shunzhao Sui, Bin Liu, Peifang Chong

**Affiliations:** 1 College of Forestry, Gansu Agricultural University, Lanzhou 730070, China; wx221069@163.com (X.W.); gin_zhenger@163.com (Y.Z.); vivi.hr@163.com (R.H.); 2 Key Laboratory of Agricultural Biosafety and Green Production of Upper Yangtze River (Ministry of Education), Chongqing Engineering Research Center for Floriculture, College of Horticulture and Landscape Architecture, Southwest University, Chongqing 400715, China; sszcq@swu.edu.cn; 3 College of Landscape and Horticulture, Yunnan Agricultural University, Kunming 650201, China; 2025064@ynau.edu.cn

**Keywords:** strigolactones, *DWARF27* gene, wintersweet, shoot branching

## Abstract

Strigolactones (SLs), as a class of novel plant hormones, play important roles in the regulation of plant branching. However, their function in branch development of wintersweet remains unclear. In this study, a gene involved in SLs biosynthesis, *CpD27*, was identified and isolated from wintersweet. The sequence characteristics, expression patterns, subcellular localization, and functional analysis through heterologous expression in *Arabidopsis thaliana* were investigated. Multiple sequence alignment showed that CpD27 contains the conserved D27 protein domain DUF4033. Quantitative real-time PCR analysis revealed that *CpD27* is expressed in various vegetative organs of wintersweet, with the highest expression in leaves, followed by axillary buds. It is also expressed in all floral organs, with the highest expression level in the outer petals. *CpD27* expression is induced by hormones (ABA and ACC) and low temperature (4 °C). Subcellular localization analysis indicated that CpD27 is localized in the chloroplasts of *Arabidopsis*. Heterologous expression of *CpD27* in *Arabidopsis* delayed bolting. The number of both rosette branches and cauline branches in transgenic plants was reduced compared with wild-type plants. In addition, the expression of *AtBRC1* was significantly upregulated in transgenic lines, suggesting that *CpD27* has a function similar to that of its homolog in *Arabidopsis*. Overall, these results indicate that *CpD27* plays a conserved role in the SLs-mediated branching pathway, which regulates branch development in wintersweet. This study provides a molecular and theoretical basis for further understanding branch development in wintersweet.

## 1. Introduction

Wintersweet (*Chimonanthus praecox* (L.) Link) is a valuable ornamental plant species native to China and is a perennial deciduous shrub. Owing to its unique winter flowering period, bright yellow flowers, and strong fragrance, it is regarded as a rare and excellent winter ornamental plant and is highly favored by landscape architects and horticultural enthusiasts [1]. Wintersweet is widely used in landscape greening, courtyard beautification, potted plant production, and as a winter cut flower [2]. As an important ornamental species, current research on wintersweet has mainly focused on the molecular mechanisms of flower development [3,4], floral scent [5], volatile compounds [4], abiotic stress responses [6], and the regulation of flower color and flavonoid biosynthesis [7]. However, branch architecture, including branch shape and structure, plays a crucial role in determining its ornamental and economic value [8]. Therefore, investigating the branching habit of wintersweet is of significant theoretical and practical importance.

Plant branching is a key determinant of plant architecture, affecting canopy structure as well as the number and yield of flowers and fruits [9]. In ornamental plants, branching patterns directly influence aesthetic value and consumer preference. In higher plants, branching is determined by the shoot apical meristem (SAM), formed during embryogenesis, and the axillary meristems (AMs), formed during post-embryonic development. The SAM establishes the primary axis, whereas branching architecture is controlled by Ams [10,11]. The branching process generally involves two developmental stages: the formation of AMs in leaf axils and the subsequent outgrowth of axillary buds. Thus, the extent of branching depends not only on the establishment of axillary meristems but also on their subsequent activity and growth.

In general, plant branching is initiated by the outgrowth of axillary buds [12]. This process is regulated by complex interactions among genetic, such as the expression of the signal integrator gene *BRANCHED1* (*BRC1*), hormonal, and environmental factors [13]. Environmental signals are integrated through long-distance signaling networks, in which plant hormones play central roles [12]. Auxin (indole-3-acetic acid, IAA), the first hormone identified to regulate branching, is synthesized in the shoot apex and young leaves and transported basipetally in a polar manner [14,15]. However, auxin does not directly enter axillary buds but regulates bud outgrowth indirectly via secondary messengers. Cytokinins (CK), the first identified secondary messengers, are synthesized throughout the plant, with roots serving as the primary source [16,17,18,19]. Root-derived CK is transported acropetally through the xylem and directly promote axillary bud growth, while buds can feedback-regulate CK export from roots. In addition, studies on branching mutants, such as *more axillary growth* (*max*) in *Arabidopsis*, *ramosus* (*rms*) in pea (*Pisum sativum*) [20,21,22], and *decreased apical dominance* (*dad*) in petunia (*Petunia hybrida*), have revealed that strigolactones (SLs), a class of carotenoid-derived hormones, act as another key regulator mediating auxin-dependent control of axillary meristem growth [23].

Strigolactones (SLs), a recently identified class of plant hormones, play essential roles in regulating plant branching by inhibiting axillary bud outgrowth [23]. SLs are derived from carotenoid cleavage and are mainly synthesized in roots, although small amounts are also produced in aerial tissues [12]. SLs biosynthesis involves a series of enzymatic reactions: *DWARF27* (*D27*) catalyzes the isomerization of all-*trans*-β-carotene to 9-*cis*-β-carotene in plastids; *CAROTENOID CLEAVAGE DIOXYGENASE 7* (*CCD7*) cleaves 9-*cis*-β-carotene to produce 9-*cis*-β-apo-10′-carotenal and β-ionone [24,25]; subsequently, *CAROTENOID CLEAVAGE DIOXYGENASE 8* (*CCD8*) converts 9-*cis*-β-apo-10′-carotenal into the precursor carlactone through a series of oxidation and rearrangement reactions [26]. Carlactone is then transported to the cytoplasm, where it is further modified by the cytochrome P450 monooxygenase *MORE AXILLARY GROWTH 1* (*MAX1*) through oxidation, cyclization, and functional group modification to produce SLs and their derivatives [27,28].

*D27* functions in the first committed step of SLs biosynthesis. Lin et al. [29] first demonstrated that rice *D27* participates in SLs biosynthesis, and the rice *d27* mutant exhibits dwarfism, increased tillering, and enhanced polar auxin transport [30]. Waters et al. identified the *Arabidopsis D27* gene and performed phylogenetic analyses across land plants, green algae, and cyanobacteria, showing that the three *Arabidopsis D27* homologs are distributed among Clades I–III. The *Arabidopsis d27* mutant similarly displays increased branching, and *D27* was confirmed to act upstream of *AtMAX1* in SLs biosynthesis. To date, *D27* gene family members have been identified and characterized in several species, including soybean, saffron (*Crocus sativus*), grape (*Vitis vinifera*), longan (*Dimocarpus longan*), sugarcane (*hybrids of Saccharum* spp.), wheat (*Triticum aestivum*), daylily (*Hemerocallis fulva*), woodland strawberry (*Fragaria vesca*), and *Nervilia fordii* [31,32,33,34].

Previous studies have shown that exogenous strigolactones inhibit axillary bud outgrowth in wintersweet [1]. However, the molecular mechanism by which the SLs biosynthetic gene *D27* regulates SLs production and subsequently controls axillary bud outgrowth in wintersweet remains unclear. In this study, the *CpD27* gene was identified and isolated from wintersweet. Sequence and phylogenetic analyses indicated that *CpD27* is an ortholog of *D27*. Expression analysis showed that *CpD27* is downregulated during branching and is expressed in roots, stems, leaves, shoot apices, axillary buds and floral organs. Overexpression of *CpD27* in *Arabidopsis thaliana* delayed bolting. The number of both rosette branches and cauline branches in transgenic *Arabidopsis* was re-duced compared with wild-type plants. These findings provide new insights into the role of *CpD27* in regulating lateral branch development in wintersweet and establish a theoretical basis for elucidating the molecular mechanisms underlying branching in this species.

## 2. Results

### 2.1. Identification of the D27 Gene Homologue from Wintersweet

Total RNA was extracted from wintersweet leaves, and first-strand cDNA was synthesized from the extracted RNA for gene cloning. The coding sequence of *CpD27* was initially identified from the wintersweet transcriptome database, the full length cDNA of 1073 bp and further verified using the wintersweet genome database [35,36]. The completeness of the CpD27 protein sequence was verified using NCBI BlastP. Sequence alignment indicated that the CpD27 protein is highly conserved with its homologs. Sequence analysis revealed a 71 bp 5′UTR (5′ untranslated region) and a 144 bp 3′UTR (3′ untranslated region), and *CpD27* contains a complete open reading frame of 858 bp, encoding a protein of 285 amino acids, with a molecular weight of 31,903.24 kDa and a pI of 9.00. Subcellular localization prediction using WOLF PSORT indicated that CpD27 is primarily localized in chloroplasts.

**Figure 1:**
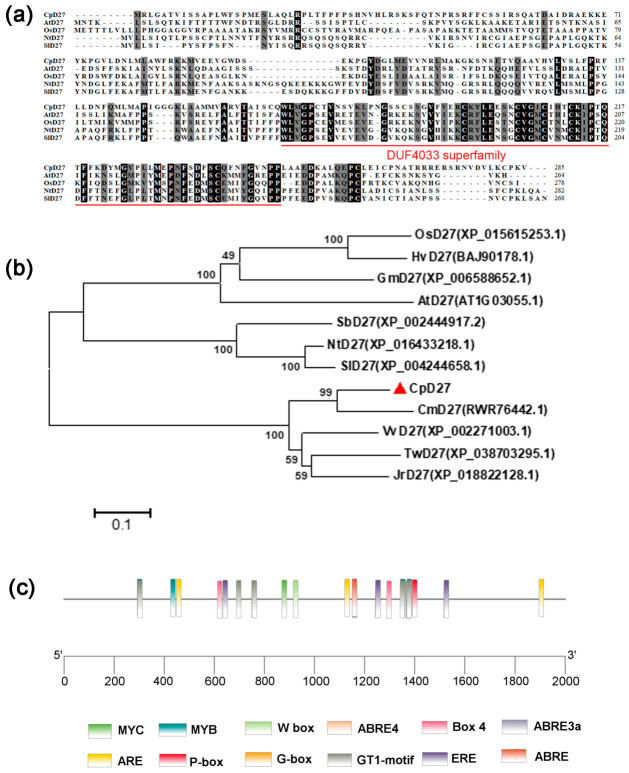
Characterization of CpD27. (**a**) Multiple sequence alignment of CpD27 with D27 proteins from *Arabidopsis thaliana* (AT1G03055.1), *Oryza sativa* (XP_015615253.1), *Nicotiana tabacum* (XP_016433218.1), and *Solanum lycopersicum* (XP_004244658.1). The red line indicates the conserved DUF4033 superfamily domain of D27. Identical amino acids are shown in black, while similar amino acids are indicated in gray. (**b**) Phylogenetic analysis of D27 proteins. The neighbor-joining (NJ) method in MEGA 6.0 software was used to construct the phylogenetic tree with 1000 bootstrap replicates. CpD27 (wintersweet) is highlighted with a red triangle. The accession numbers of protein sequences used for phylogenetic analysis are listed in Table S2. (**c**) Cis-acting regulatory elements in the CpD27 promoter.

Multiple sequence alignment using BioEdit showed that CpD27 shares high similarity with D27 proteins from *Arabidopsis thaliana*, rice, tobacco, and tomato, all containing the conserved DUF4033 superfamily domain (Figure 1a). Phylogenetic analysis using MEGA 6.0 included CpD27 and homologs from *A. thaliana* (AtD27), *Oryza sativa* (OsD27), *Solanum lycopersicum* (SlD27), *Glycine max* (GmD27), *Sorghum bicolor* (SbD27), *Nicotiana tabacum* (NtD27), *Hordeum vulgare* (HvD27), and the closest NCBI homologs, including *Cinnamomum micranthum* (CmD27), *Vitis vinifera* (VvD27), *Tripterygium wilfordii* (TwD27), and *Juglans regia* (JrD27). The phylogenetic tree indicated that CpD27 clusters with CmD27 and is most closely related to woody plants such as *C. camphora*, grape, *T. wilfordii*, and walnut (Figure 1b).

Meanwhile, we analyzed the 2000 bp upstream promoter region of the *CpD27* gene. Cis-acting regulatory elements were identified using the PlantCARE online tool. The results showed that the *CpD27* promoter contains light-responsive elements (Box4, G-box, and GT1-motif), a meristem-specific expression element (CAT-box), hormone-responsive elements including ABA-responsive element (ABRE), ethylene-responsive element (ERE), and gibberellin-responsive element (P-box), as well as a WRKY transcription factor binding site (W-box), an anaerobic-responsive element (ARE), and MYC and MYB elements associated with drought, high salinity, and low temperature responses (Figure 1c and Table S3).

### 2.2. Tissue Specificity of CpD27 Expression

**Figure 2:**
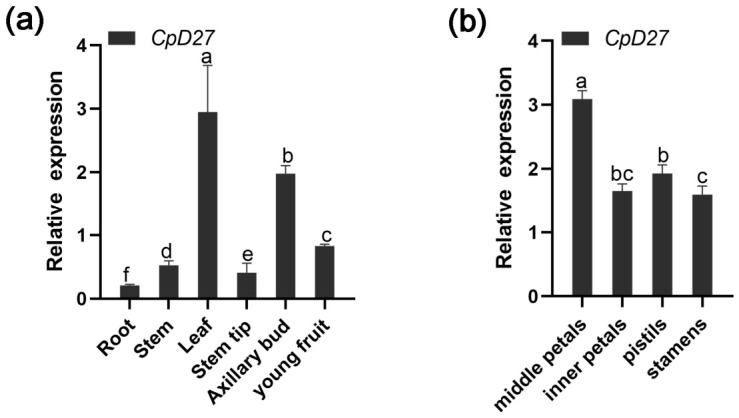
Expression pattern of *CpD27* gene. (**a**) Expression of *CpD27* in different organs of wintersweet. (**b**) Expression of *CpD27* in various floral organs of wintersweet. *CpActin* was used as the internal reference gene. Each group included three biological replicates and three technical replicates, and data are presented as mean ± standard deviation (SD). Different lowercase letters above the bars indicate significant differences (*p* < 0.05).

To further investigate the function of *CpD27* in wintersweet, its expression patterns were analyzed in roots, stems, leaves, shoot apices, axillary buds, and floral organs. *CpD27* was expressed in all organs, with highest levels in leaves, followed by axillary buds (Figure 2a). *CpD27* was expressed in all floral organs, with the highest expression level observed in the outer petals (Figure 2b), suggesting a role in leaf morphogenesis, axillary bud and floral development.

### 2.3. Expression Patterns of CpD27 Under Abiotic Stresses and Hormone Treatments

**Figure 3:**
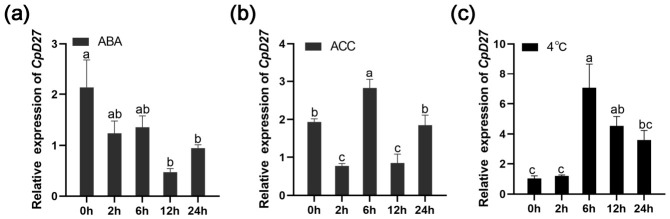
Expression analysis of *CpD27* under different plant hormone treatments and abiotic stress conditions in wintersweet seedlings. (**a**) 50 µM ABA treatment; (**b**) 100 µM ACC treatment; (**c**) 4 °C cold treatment. Different lowercase letters indicate significant differences among different time points within the same treatment (*p* < 0.05).

To investigate the responsiveness of *CpD27* to plant hormones and abiotic stresses, 6-leaf-stage wintersweet seedlings were treated with 50 µM ABA, 100 µM ACC (a precursor of ethylene biosynthesis), and low temperature at 4 °C. Gene expression levels were then measured at different time points. As shown in Figure 3, *CpD27* expression was downregulated following ABA treatment, reaching its lowest level at 12 h (Figure 3a). After ACC treatment, *CpD27* showed differential expression and reached its highest level at 6 h (Figure 3b). Under 4 °C treatment, *CpD27* expression first increased and then decreased, peaking at 6 h (Figure 3c). These results indicate that *CpD27* expression is responsive to ABA, ACC, and low temperature treatments, suggesting that the gene may be transcriptionally regulated by these signals. Further functional studies are needed to determine whether *CpD27* directly participates in hormone signaling or stress response pathways.

### 2.4. CpD27 Is Localized to the Plastid

**Figure 4:**
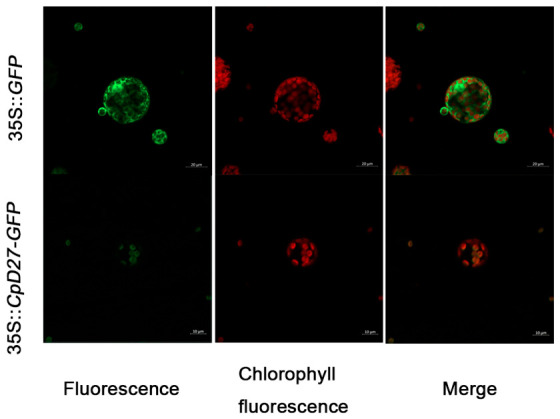
Subcellular localization analysis of CpD27. Subcellular localization analysis of GFP-tagged CpD27. The GFP-fused CpD27 construct was expressed in *Arabidopsis thaliana* protoplasts. The 35S::GFP construct was used as a control. Green indicates GFP fluorescence (**left panel**), red indicates chlorophyll autofluorescence (**middle panel**), and yellow represents the merged signals (**right panel**).

The constructed pCAMBIA1300-*CpD27* plasmid and the positive control 35S::GFP were individually introduced into *Agrobacterium tumefaciens* GV3103. Subcellular localization of the CpD27 protein was examined by transient expression in *Arabidopsis thaliana* protoplasts. Confocal laser scanning microscopy showed that the CpD27–GFP fusion protein was localized in the chloroplasts of protoplasts, whereas the control GFP signal was distributed throughout the entire protoplast (Figure 4). These results suggest that CpD27 is likely localized in chloroplasts or plastids of wintersweet roots.

### 2.5. Overexpression Arabidopsis of CpD27

A 35S::*CpD27* construct was generated and transformed into wild-type *Arabidopsis thaliana*. Nineteen transgenic lines were obtained through hygromycin selection and PCR verification. RNA extracted from leaves of positive lines was reverse-transcribed to cDNA and analyzed by qRT-PCR with *AtActin* as internal control and WT as negative control. *CpD27* was expressed in all transgenic lines but undetectable in WT (S1). After expression level analysis, three transgenic lines with high, medium, and low expression levels (lines 4, 5, and 9) were selected and designated as OE1, OE2, and OE3, respectively, for subsequent phenotypic analysis.

**Figure 5:**
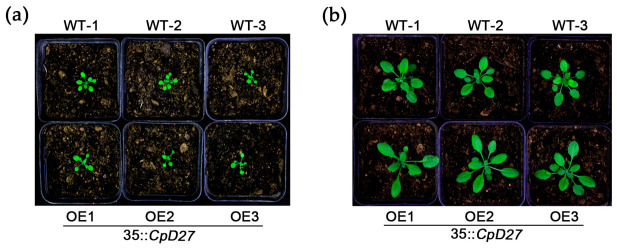
*CpD27* overexpresses the phenotype of *Arabidopsis* seedlings. (**a**) Transgenic lines and WT (WT 1–3) seedlings 10 days after transplanting; (**b**) Transgenic lines and WT (WT 1–3) seedlings 3 weeks after transplanting.

Under long-day conditions, 10-day-old transgenic seedlings had sparser leaves than WT (Figure 5a). Three weeks after transplanting, transgenic plants displayed larger leaves (Figure 5b).

**Figure 6:**
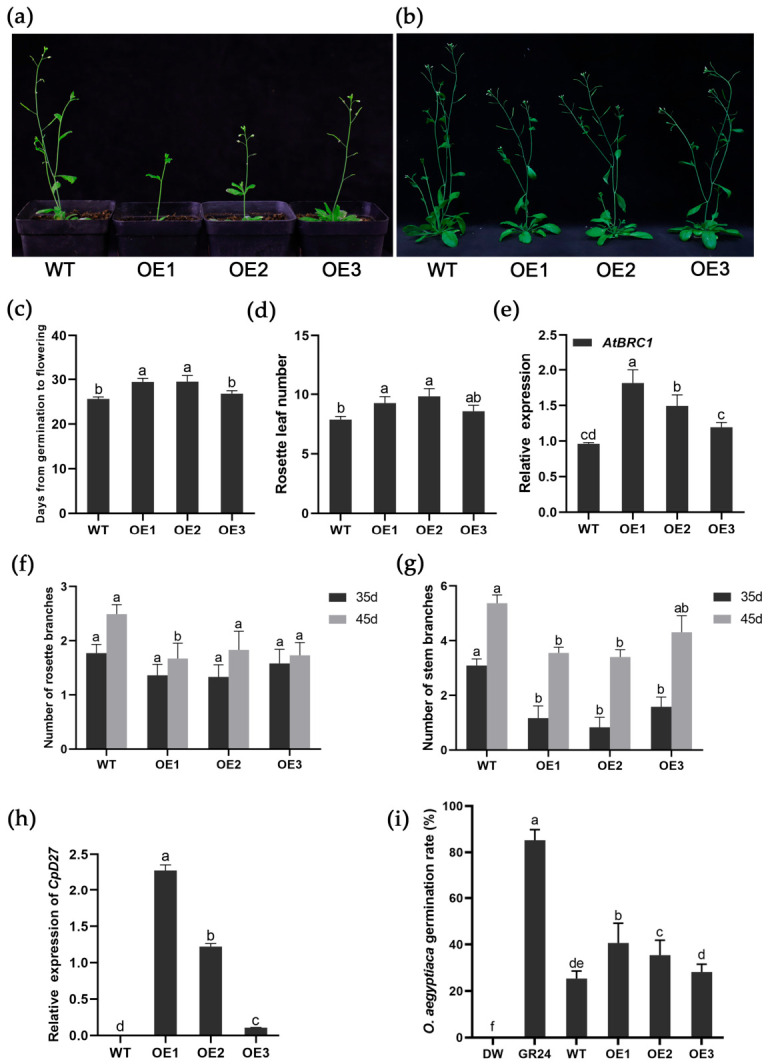
Phenotype of *CpD27* overexpression lines. (**a**) Rosette branching phenotype at 35 days after transplantation. (**b**) Rosette branching phenotype at 45 days after transplantation. (**c**) Bolting days required for overexpression lines and WT plants. (**d**) The number of rosette leaves of overexpression plants and WT plants, bolting 1 cm. (**e**) The expression level of *AtBRC1* gene in the overexpression line, and WT was the control. (**f**) Number of rosette branches of overexpression lines and WT plants. (**g**) Number of stem branches in overexpression lines and WT plants. (**h**) The level of *CpD27* expression in the overexpression lines, WT is the control. The reference gene in *Arabidopsis* is *AtActin* (Gene ID: 823805). (**i**) The mixed extract of root and stem of *CpD27* overexpression plants induces the germination rate of *Orobanche* seeds. DW: deionized water as a negative control, and GR24 as a positive control. Different lowercase above the bars indicates significant differences (*p* < 0.05).

Under long-day conditions, we observed that bolting was delayed in transgenic plants, WT bolted 25.64 ± 0.44 days after transplant, while OE1, OE2, and OE3 bolted at 29.45 ± 0.79, 29.50 ± 1.38, and 26.82 ± 0.67 days, respectively (Figure 6a,c). Rosette leaf numbers were higher in transgenic plants (WT: 7.88 ± 0.26; OE1: 9.27 ± 0.54, OE2: 9.83 ± 0.65, OE3: 8.85 ± 0.50) (Figure 6d).

Rosette and stem branch numbers were recorded at 35 and 45 days. *CpD27* overexpression reduced rosette and stem branching. At 35 days, the rosette branches of the transgenic lines were fewer than those of the WT, but the differences did not reach statistical significance (WT: 1.77 ± 0.16; OE1: 1.36 ± 0.20; OE2: 1.33 ± 0.23; OE3: 1.58 ± 0.26). The stem branches of the transgenic lines were significantly fewer than those of the WT (WT: 3.09 ± 0.24); the values for the three transgenic lines OE1, OE2, and OE3 were 1.77 ± 0.44, 0.83 ± 0.37, and 1.58 ± 0.36, respectively. At 45 days, the rosette branches of the transgenic lines were fewer than those of the WT. The OE1 line showed significantly fewer rosette branches than the WT (WT: 2.49 ± 0.18; OE1: 1.67 ± 0.28), while the OE2 and OE3 lines did not show statistically significant differences (OE2: 1.83 ± 0.35; OE3: 1.73 ± 0.24). Except for the OE3 line (OE3: 4.31 ± 0.60), the stem branches of the OE1 and OE2 lines were significantly fewer than those of the WT (WT: 5.37 ± 0.30; OE1: 3.55 ± 0.55; OE2: 3.40 ± 0.27) (Figure 6f,g).

*BRC1*, a TCP family CYC1 subclade member, inhibits lateral branch elongation in maize, rice, tomato, and pea, integrating branching signals [37,38]. Expression analysis showed higher *AtBRC1* levels in *CpD27-OE* lines than WT (Figure 6e).

Since SLs can induce the germination of *Orobanche* seeds, using plant tissue extracts to induce *Orobanche* seed germination is commonly regarded as a simple and reliable method for the preliminary detection of SLs in target species. Therefore, in this study, extracts from transgenic *Arabidopsis* were used to induce germination of *Orobanche* seeds for preliminary analysis of strigolactone (SLs) content. Germination assays using root-stem mixed extracts from overexpression lines induced higher seed germination than WT extracts (Figure 6i), consistent with elevated *CpD27* expression (Figure 6h). These results indicate that *CpD27* suppresses rosette branching via increased SLs levels and may involve downstream transcriptional regulation of *BRC1*.

## 3. Discussion

With the identification of numerous branching mutants, key components of the SLs pathway, including *D27*, *MAX3*, *MAX4*, *MAX1*, *MAX2*, *D14*, and *D53*, have been successively characterized. SLs are synthesized from carotenoid precursors, and *D27* encodes an iron-containing β-carotene isomerase that plays a crucial role in SLs biosynthesis. Specifically, *D27* (*AtD27*) catalyzes the conversion of all-trans-β-carotene to 9-cis-β-carotene, providing the substrate for subsequent cleavage by *CCD7* to produce SLs [27,29]. To investigate the role of *D27* in regulating branching via the SLs pathway in Wintersweet, the *CpD27* gene was isolated and characterized. Sequence alignment revealed that *CpD27* shares high similarity with homologs from *Arabidopsis thaliana*, rice, tomato, and tobacco, all containing the conserved DUF4033 domain (Figure 1a). Phylogenetic analysis further showed that CpD27 clusters closely with homologs from woody species such as *Cinnamomum camphora*, *grape*, *Tripterygium wilfordii*, and walnut, forming a clade with CmD27 (Figure 1b), suggesting evolutionary conservation of function.

Gene expression patterns often reflect gene function. Previous studies have shown that *D27* exhibits species-specific expression patterns. For example, *OsD27* is mainly expressed in panicles and axillary buds in rice [29], *DgD27* is predominantly expressed in stems and shoot apices in *chrysanthemum* [8], *ScD27* shows high expression in tiller buds, axillary buds, and shoot apices in sugarcane [39], and *TaD27* is mainly expressed in leaves, leaf sheaths, and stems in wheat [40]. In the present study, *CpD27* was expressed in all examined organs, including roots, stems, leaves, shoot apices, axillary buds and floral organs, with relatively higher expression in leaves, axillary buds and the outer petals. These results suggest that *CpD27* may play a role in leaf morphogenesis, axillary bud and floral development in wintersweet. It is interesting to note that during hormone treatment, we observed fluctuating expression of *CpD27* following ACC application: expression levels decreased at 2 h and 12 h, while reaching a peak at 6 h. This likely reflects a dynamic and transient response to ethylene signaling. It is possible that the gene is rapidly induced upon ACC application, but subsequent negative feedback or adaptive mechanisms temporarily suppress its expression at 2 h. The peak at 6 h may represent a secondary or sustained activation phase, followed by another decline at 12 h as the signal attenuates. However, the underlying mechanisms require further investigation. Subcellular localization analysis indicated that CpD27 is mainly localized in chloroplasts of *Arabidopsis* protoplasts, implying that SLs biosynthesis mediated by *CpD27* may occur in plastids of roots as well as in chloroplasts of leaves and axillary buds in wintersweet, although the precise localization requires further investigation.

*D27* was first identified as a key enzyme in SLs biosynthesis in rice [29] and later confirmed in *Arabidopsis thaliana* [27,41]. Mutations in *D27* result in dwarfism and increased branching in both rice and *Arabidopsis*, and these phenotypes can be rescued by treatment with the SLs analog GR24. Functional conservation of *D27* has also been demonstrated in other species; for instance, *chrysanthemum DgD27* can rescue the phenotype of *Arabidopsis* mutants [8], while RNAi-mediated silencing of *TaD27* increases tillering in wheat [40]. To further elucidate the role of *CpD27* in lateral branch development, the *CpD27* gene was cloned into a plant expression vector and overexpressed in *Arabidopsis thaliana*. Transgenic lines with high, medium, and low expression levels (T3 generation) were selected based on PCR identification and qRT-PCR analysis for phenotypic characterization. Under long-day conditions, transgenic seedlings exhibited phenotypic differences compared with wild-type (WT) plants, including sparser leaves at early stages (10 days after transplantation) and larger leaves after three weeks. These observations suggest that *CpD27* may also be involved in regulating leaf morphogenesis, which warrants further investigation.

Bolting represents the transition from vegetative to reproductive growth in plants and marks the initiation of inflorescence development in *Arabidopsis*. Rosette leaf number is also an important indicator of vegetative growth status. In this study, the time required for bolting and the number of rosette leaves were recorded when the bolting stem reached 1 cm. The results showed that *CpD27* overexpressing plants exhibited delayed bolting and increased rosette leaf numbers compared with WT plants. Furthermore, analysis of rosette and stem branching at 35 and 45 days revealed that transgenic plants had fewer rosette and stem branches than WT plants. Even the lowest expressing OE3 line exhibited significantly fewer stem branches than the WT at 45 days. This indicates that even a relatively low level of *CpD27* activity is sufficient to partially inhibit branching (Figure 6). This is consistent with the conclusions obtained from species such as *Arabidopsis* [41], *chrysanthemum* [8], wheat [40], rice [30], and sugarcane [9], further supporting the functional conservation of the *D27* gene in both monocot and dicot plants. However, in addition to the branching-reducing effect of *CpD27*, which is consistent with previous studies, overexpression of *CpD27* also delayed bolting and increased rosette leaf number in *Arabidopsis*. It remains unclear whether the observed pleiotropic phenotypes are a direct effect of *CpD27* itself or an indirect consequence of altered SLs homeostasis. Given that *CpD27* is a key enzyme in the SLs biosynthesis pathway, we speculate that the observed pleiotropic phenotypes are likely mediated by changes in endogenous SLs levels or SLs signaling, and this part requires further validation. Moreover, instead of directly measuring SLs levels in the overexpression lines using high-performance liquid chromatography-tandem mass spectrometry (HPLC-MS/MS), we opted for the most basic *Orobanche* seed germination test, which constitutes a limitation of this study and needs to be addressed in future work.

Taken together, these results indicate that *CpD27* functions similarly to *D27* homologs in other plant species, acting as a key regulator in the SLs biosynthetic pathway to control axillary bud development and branching. This study provides new insights into the molecular mechanisms underlying branching regulation in wintersweet and lays a foundation for further functional analysis and genetic improvement of plant architecture in ornamental species. Future research could focus on identifying upstream transcriptional regulators that bind to the *CpD27* promoter, exploring potential interacting proteins of *CpD27* with other SLs biosynthetic or signaling components, and profiling downstream gene expression changes. Addressing these questions will further elucidate the molecular network controlling shoot branching and facilitate genetic improvement of plant architecture in ornamental crops.

## 4. Materials and Methods

### 4.1. Plant Materials and Growth Conditions

Wintersweet ‘Suxin’ seeds were collected from Southwest University (Chongqing, China). After treatment with 98% sulfuric acid for 30 min, seeds were rinsed thoroughly, surface-sterilized, and sown in pots containing peat:vermiculite (3:1, *v*/*v*). Plants were grown at 25 °C under a 16 h light/8 h dark photoperiod in the Floriculture Laboratory.

Wild-type Arabidopsis thaliana Columbia-1 (col-1) (*Arabidopsis thaliana* (L.) Heynh) was maintained in the Floriculture Laboratory of Southwest University and used as a control.

### 4.2. Cloning of CpD27 Genes

Total RNA was extracted from leaves of wintersweet seedlings using the Aidlab RNA Rapid Extraction Kit (Aidlab, Beijing, China) according to the manufacturer’s instructions. First-strand cDNA was synthesized using the PrimeScript RT-PCR Kit (TaKaRa, Dalian, China) following the manufacturer’s protocol. The amplification program for the *CpD27* gene was as follows: pre-denaturation at 95 °C for 5 min; followed by denaturation at 95 °C for 30 s, annealing at 58 °C for 30 s, and extension at 72 °C for 50 s; with a final extension at 72 °C for 10 min. The PCR products were separated by 1% agarose gel electrophoresis, and the target DNA fragments were recovered using an agarose gel DNA extraction kit (Tiangen, Beijing, China). The purified fragments were cloned into the pMD19-T vector (TaKaRa, Dalian, China) and sequenced by Qingke Biotechnology Co., Ltd. (Chengdu, China).

Based on the published reference genome of wintersweet, approximately 2000 bp upstream of the start codon (ATG) of the target gene was extracted as the candidate promoter region. Specific primers were designed using Primer Premier 5.0 software (Table S1), and PCR amplification was performed using wintersweet genomic DNA as the template.

Amino acid sequence alignment was performed using BioEdit version 7.0.5 (Hall, 1999) with the ClustalW algorithm. A phylogenetic tree was constructed using the neighbor-joining (NJ) method in MEGA 6.0 with 1000 bootstrap replicates. Amino acid sequences of D27 proteins from other plant species used for alignment and phylogenetic analysis were obtained from the National Center for Biotechnology Information (NCBI: http://www.ncbi.nlm.nih.gov/), accessed on 10 May 2021. Analysis of cis-acting elements of the *CpD27* promoter was performed using PLANTCARE (http://bioinformatics.psb.ugent.be/webtools/plantcare/html/, accessed on 23 April 2026). The subcellular localization of the D27 protein was predicted using the WOLF PSORT online analysis software (https://wolfpsort.hgc.jp, accessed on 21 May 2020).

### 4.3. Subcellular Localization

To determine the subcellular localization of CpD27, the ORF of *CpD27* without the stop codon was cloned into the pCAMBIA1300 vector using SacI and BamHI restriction sites, and the resulting construct was named pCAMBIA-1300-*CpD27*. The correct construct was verified by PCR and double-enzyme digestion and then used for subsequent experiments. Agrobacterium strain GV3101 was used to introduce 35S:CpD27-GFP and the empty vector into *Arabidopsis thaliana* protoplasts, respectively. Protoplast transformation was performed using the *Arabidopsis* protoplast preparation and transformation kit (Coolaber, Beijing, China) according to the manufacturer’s instructions, and green fluorescent protein (GFP) signals were observed using a confocal microscope (Tokyo, Japan). The primers used for plasmid construction are listed in Table S1.

### 4.4. Vector Construction and Plant Transformation

The *CpD27* gene was introduced into an entry vector via a BP reaction using the Gateway system and subsequently recombined into the plant expression vector pGWB551 through an LR reaction. The resulting recombinant plasmid was transformed into *Escherichia coli* and verified by colony PCR. Plasmids with correct sequences were extracted and then introduced into *Agrobacterium tumefaciens* strain GV3101. The bacterial suspension was cultured for approximately 15 h until the OD_600_ reached 0.8–1.0, and then used for *Arabidopsis thaliana* transformation. The constructed 35S:*CpD27*-pGWB551 vector was introduced into wild-type plants using the floral dip method.

The harvested T0 seeds of transgenic *Arabidopsis* were dried in an oven at 37 °C for storage. The seeds were surface-sterilized with 2% sodium hypochlorite (NaClO) for 7 min, rinsed thoroughly with sterile water, and sown on MS medium (pH 5.8) containing hygromycin (25 mg/L) for selection. The plants were grown in a growth chamber under long-day (LD) photoperiod conditions at 22 °C. Homozygous T3 lines were used for phenotypic analysis.

### 4.5. Quantitative Real-Time PCR(qRT-PCR) Analysis

Roots, stems, shoot apices, and leaves were harvested from 2-month-old seedlings, while flowers were sampled at anthesis from 5-year-old plants. All organs were snap-frozen in liquid nitrogen and stored at −80 °C until RNA extraction.

After cis-acting element analysis of the *CpD27* gene promoter, based on the distribution and classification of promoter elements, six-leaf-stage wintersweet seedlings of uniform growth were selected. The leaves were sprayed with 50 μM ABA [2] and 100 μM ACC [42], respectively, until droplets began to drip. Samples were collected at 0, 2, 6, 12, and 24 h after treatment, immediately flash-frozen in liquid nitrogen, and then stored at −80 °C for later use. For each replicate, two top leaves were taken from each seedling, and each treatment consisted of three biological replicates. Total RNA was extracted from the samples, and quantitative real-time PCR (qRT-PCR) was performed to analyze the expression pattern of the *D27* gene in wintersweet. qRT-PCR was performed using a Bio-Rad CFX96 real-time system and Ssofast EvaGreen Supermix (BIO-RAD, Hercules, CA, USA). The primers qRTCpD27-F/R are listed in Supplementary Table S1, and the *CpActin* gene was used as an internal control. All primers were designed using Primer Premier 6.0 software. qRT-PCR was carried out under the following conditions: 98 °C for 3 min, followed by 40 cycles of 95 °C for 5 s, 58 °C for 5 s, and 72 °C for 5 s, and a melt curve from 65 °C to 95 °C. Three biological replicates and three technical replicates were used for each sample. Gene expression levels were analyzed using the 2^−ΔΔCT^ method.

### 4.6. Orobanche Seed Germination Assay

Seeds of *Orobanche aegyptiaca* were surface-sterilized with 75% ethanol and 1% NaClO, then rinsed thoroughly with sterile water and air-dried. Sterilized glass fiber filter papers were punched into 6 mm disks, and 30–50 seeds were evenly placed on each disk. After transgenic plants were grown for 45 days, whole plants were harvested, flash-frozen in liquid nitrogen, and stored at −80 °C. Samples were freeze-dried, ground into powder, and passed through a sieve. Then, 100 mg of powder was extracted with methanol using ultrasonication for 30 min. After centrifugation, the supernatant was collected and diluted to 10 mg/mL. A total of 20 µL of extract was applied onto each disk. The disks were sealed and incubated at 25 °C in darkness for 10 days. Germination rates were then recorded under a stereomicroscope. A 10 µM GR24 solution was used as the positive control, and sterile water as the negative control. Each treatment included five biological replicates.

### 4.7. Statistical Analysis

All experiments were replicated three times to confirm the results. The data presented are mean ± standard error from a typical single experiment. ANOVA was conducted, followed by a Duncan’s test. Different letters indicate significant differences (*p* < 0.05) between different treatments.

## 5. Conclusions

This study systematically analyzed the molecular characteristics, expression patterns, and gene function of *CpD27* in wintersweet. CpD27 contains the conserved D27 protein domain DUF4033 and is localized in the chloroplasts. It is expressed in various organs of wintersweet, with relatively high expression levels in vegetative organs such as leaves and axillary buds, and the highest expression observed in the outer petals among floral organs. *CpD27* expression is induced by ABA, ACC, and low temperature (4 °C). Overexpression of *CpD27* reduced both rosette and cauline branching in *Arabidopsis thaliana*. In summary, these findings suggest that *CpD27* plays a conserved role in the regulation of branching, providing a potential target for genetic improvement and molecular breeding of plant architecture in wintersweet.

## Data Availability

Data are contained within the article and Supplementary Materials.

## Supplementary Materials

The following supporting information can be downloaded at: https://www.mdpi.com/article/10.3390/plants15121926/s1, Figure S1: *CpD27* overexpression identification of the relative expression level of *CpD27* gene in *Arabidopsis*. 1–19: *CpD27* transgenic lines; WT: wild-type *Arabidopsis*; Table S1: Primers were used for amplification of wintersweet genes; Table S2: The accession numbers of protein sequences used for phylogenetic analysis; Table S3: Analysis of regulatory elements of *CpD27* promoter.
