# Supplementary material for: The DWARF27 Gene from Wintersweet (Chimonanthus praecox) Encodes an All-Trans/9-cis-β-Carotene Isomerase, Which Regulates Shoot Branching in Arabidopsis
Source: Plants (Basel). 2026 Jun 22;15(12):1926. doi: 10.3390/plants15121926 (PMC13306238; doi:10.3390/plants15121926)
Supplement: Supplementary file 1 [file plants-15-01926-s001.zip › plants-4336579-supplementary.pdf]

Table S1. Primers were used for amplification of wintersweet genes

Primer sequences (5'-3')	Primer sequence (5'-3')	Purpose of primers	Annealing temperatures°C
CpD27-F	CACTTCAGATCATGCGTTTGG	Cloning primers for CpD27	58
CpD27-R	GGAGGACATTCTACACCTTTGG		
V-CpD27-F	ATGCGTTTGGGCGCCACTGTTATC	Full-length amplification	55
V-CpD27-R	CTACACCTTTGGGCACTTCAAGAC		
BP-CpD27-F	GGGGACAAGTTTGTACAAAAAAGCAGGCT *att*B2 ATGCGTTTGGGCGCCACTGTTAT	Primers for BP reaction	
BP-CpD27-R	GGGGACCACTTTGTACAAGAAAGCTGGGT *att*B2 CTACACCTTTGGGCACTTCAAGA		
L- CpD27-F	GGGGTACCATGCGTTTGGGCGCCACTGTTATC	primers for subcellular localization	58
L- CpD27-R	GCTCTAGACACCTTTGGGCACTTCAAGACATC		
qRT-CpD27-F	GCGACAGATGCGATTGACAGAG	Real-time quantification	58
qRT-CpD27-R	GCCATGAGACGGTTGACAACTT		
CpActin-F	AGGCTAAGATTCAAGACAAGG		58
CpActin-R	TTGGTCGCAGCTGATTGCTGTG		
qRT-AtBRC1-F	TTCCCAGTGATTAACCACCAT		56
qRT-AtBRC1-R	TCCGTAAACTGATGCTGCTC		
AtActin-F	GACTCAGATCATGTTTGAGACCTTT		59
AtActin-R	CCAGAGTCCAACACAATACCG		

Table S2. The accession numbers of protein sequences used for phylogenetic analysis

Species	Accession numbers
A. thaliana	AT1G03055.1
Oryza sativa	XP_015615253.1
Glycine max	XP_006588652.1
Solanum lycopersicum	XP_004244658.1
Nicotiana tabacum	XP_016433218.1
Sorghum bicolor	XP_002444917.2
Hordeum vulgare	BAJ90178.1
Cinnamomum micranthum	RWR76442.1
Tripterygium wilfordii	XP_038703295.1
Vitis vinifera	XP_002271003.1
Juglans regia	XP_018822128.1

Table S3. Analysis of regulatory elements of *CpD27* promoter

Site names	Amount	Sequence	Function of site
CAAT-box	51	CCAAT/CAAT/CCAAT	Common cis-acting elements in promoter and enhancer regions

MYC	1	CAATTG/CATGTG	Responsive to drought and ABA signals
ARE	3	AAACCA	essential regulatory element for anaerobic induction
Box 4	3	ATTAAT	light-responsive conserved DNA motifs
MYB	4	CAACAG/ TAACCA	responsive to drought, high salinity, and low temperature
P-box	1	CCTTTTG	gibberellin-responsive element
ABRE3a	1	TACGTG	involved in response to ABA and abiotic stress
W box	1	TTGACC	WRKY transcription factor-specific binding element
CAT-box	2	GCCACT	meristem expression-related cis-acting elements
G-box	2	TACGTG/ TACGTG	elements involved in light response
ERE	5	ATTTTAAA	ethylene-responsive element
ABRE4	1	CACGTA	ABA-responsive and stress-responsive elements
GT1-motif	6	GGAGATG	light-responsive element
ABRE	1	ACGTG	ABA-responsive element
TATA-box	83	TATA	The transcription start site is a core promoter element

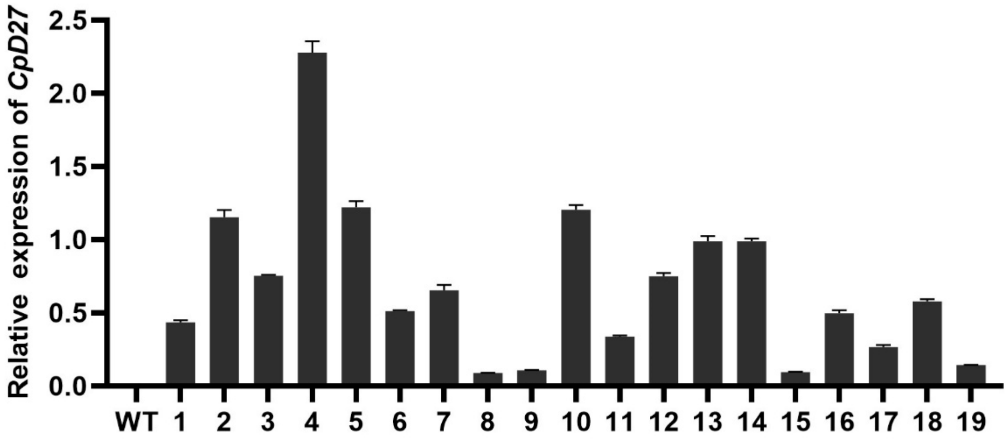

Figure S1. *CpD27* overexpression identification of the relative expression level of *CpD27* gene in *Arabidopsis*.1-19: *CpD27* transgenic lines; WT: wild-type *Arabidopsis*.
